# Acute Calcific Tendonitis of the Longus Colli: An Uncommon Cause of Neck Pain in the Emergency Department

**DOI:** 10.7759/cureus.9295

**Published:** 2020-07-20

**Authors:** Nicolas Ulloa, Jaskirat Gill, John Childress

**Affiliations:** 1 Emergency Medicine, Aventura Hospital and Medical Center, Aventura, USA

**Keywords:** acute calcific tendonitis, neck pain, sports medicine, orthopedics, emergency medicine, longus colli

## Abstract

The longus colli muscle has three major parts that originate and insert in the upper cervical and thoracic spine. It is a weak flexor of the neck, and when contracted also serves to rotate the neck to the ipsilateral side. It is innervated by the anterior rami of the C2-C6 spinal nerves and receives its blood supply from the anterior cervical and inferior thyroid arteries. In a post motor vehicle collision (MVC) patients presenting weeks later, the physician has to ensure there is no subacute fracture. Clinically, these patients present with severe anterior neck pain that will often be described as a deep pain.

A 43-year-old female with a past medical history of diabetes and prior surgical history of a tonsillectomy and uvulectomy presented with sudden onset of sore throat that woke her up from sleep at 3:00 am. Associated with the sore throat, she had hoarseness of her voice, difficulty breathing and felt that her throat was closing. She forced herself to vomit and then felt better. She denied any fevers or chills. Later, the patient mentioned that she had chronic neck pain from a prior accident and had been told that she has C5-C6 stenosis. About a week prior, she had been involved in another MVC and had some neck pain after that. Significantly, she was also on lisinopril for her hypertension (HTN). She was tolerating secretions, protecting her airway and no gross inflammation was noted on physical exam. On labs, there was no leukocytosis noted. Soft tissue neck X-ray showed reversal of the cervical lordosis, degenerative and erosive changes at C4-C5 and C5-C6, and thickening of the prevertebral soft tissues. She obtained a CT of the neck and cervical spine that showed osteophyte complexes at C4-C5 and C5-C6, as well as calcific tendonitis of the longus colli with a moderate amount of prevertebral free fluid. Neurosurgery was consulted from the ED who recommended getting an MRI of the neck, and consulting ENT as well. ENT happened to be in the ED and performed a bedside laryngoscopy that showed edema of the left arytenoid with full functionality of the vocal cords, and no signs of airway compromise. The patient was started on steroids, antibiotics, H2 blockers, and the ACE-inhibitor was discontinued due to suspected angioedema per ENT. She was also admitted to the ICU for airway monitoring. The MRI of the neck again showed calcific tendonitis of the longus colli with moderate prevertebral fluid. Meanwhile, the patient had complete resolution of her symptoms in 24 hours and she was cleared from both neurosurgery and ENT to be discharged. Acute calcific tendonitis is due to the deposition of calcium hydroxyapatite, which can cause significant pain and edema. In terms of calcific tendonitis of the longus colli muscle, this condition is frequently misdiagnosed and continues to be a challenge, especially in the emergency department. The differential diagnosis is diverse in etiology and severity, ranging from meningitis and retropharyngeal abscess to vertebral fracture and muscle strain.

## Introduction

Neck pain is a common cause of long-term disability around the world. A subset seen commonly in the emergency department (ED) is post motor vehicle collision (MVC) neck pain that is associated with whiplash [1]. Often these patients will not achieve complete resolution of their symptoms and even despite treatment, 50-85% will have a recurrence in their symptoms 1-5 years later [2]. Although previous axial pathology significantly contributes to the worsening of neck pain after an MVC, patients often fail to appreciate the link between the two [3]. In the acute setting, the most important decision is differentiating life-threatening causes of neck pain from non-life-threatening musculoskeletal causes. Of these, one rarely encountered and underdiagnosed cause in the ED is acute calcific tendonitis of the longus colli muscle.

The longus colli muscle has three major parts that originate and insert in the upper cervical and thoracic spine. It is a weak flexor of the neck and contributes to ipsilateral rotation when contracted. It is innervated by the anterior rami of the C2-C6 spinal nerves and receives its blood supply from the anterior cervical and inferior thyroid arteries [4]. Clinically these patients present with severe anterior neck pain that will often be described as a deep pain. In a post MVC patient presenting weeks later, the physician has to ensure there is no subacute fracture.

## Case presentation

A 43-year-old female with a past medical history of diabetes and prior surgical history of a tonsillectomy and uvulectomy presented with a sudden onset of sore throat that woke her up from sleep at 3:00 am. Associated with the sore throat, she had hoarseness of her voice, difficulty breathing and felt that her throat was closing. She forced herself to vomit and then felt better. She denied any fevers or chills.

Later, the patient mentioned that she had chronic neck pain from a prior accident and had been told that she has C5-C6 stenosis. About a week prior, she had been involved in another MVC and had some neck pain after that. She had also reported to be taking lisinopril.

Pertinent physical exam findings were mild erythema of the posterior oropharynx, pain on palpation along the left lateral neck and some decreased range of motion. She was tolerating secretions, protecting her airway and no gross inflammation was noted on the exam.

**Figure 1:**
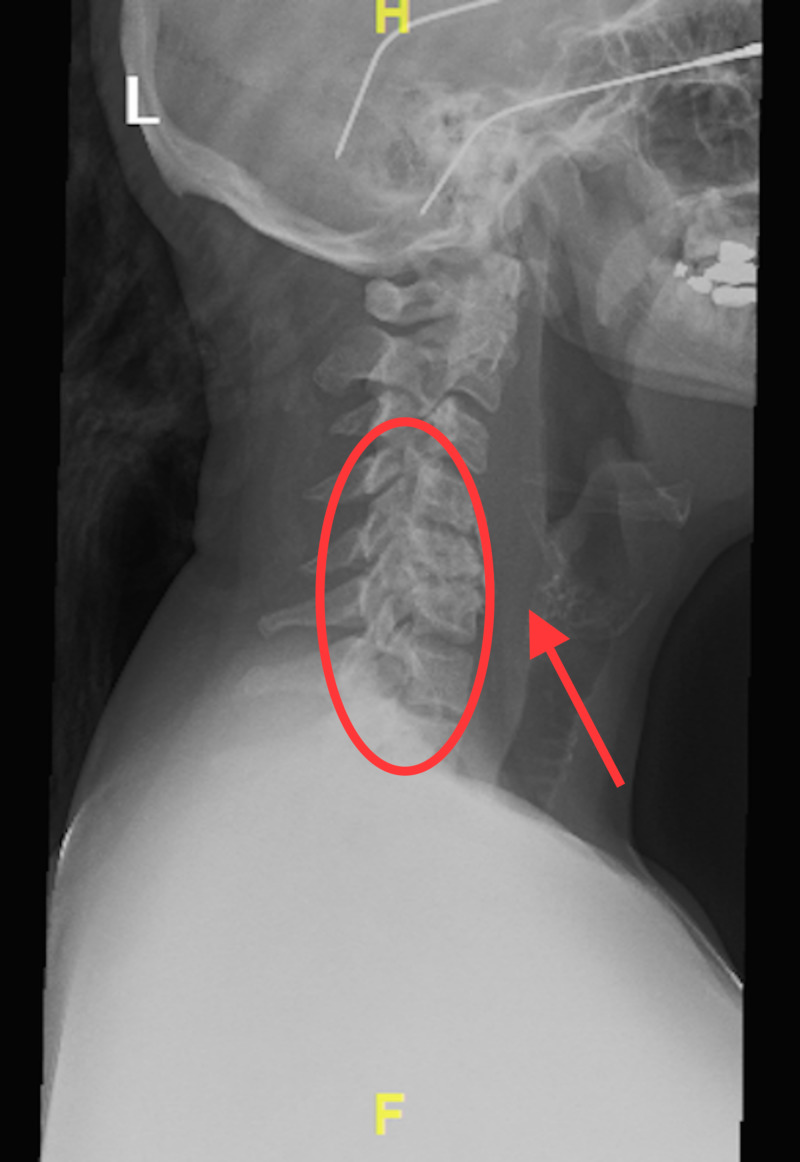
Soft tissue neck X-ray showing degenerative changes (within the oval) of the cervical spine at C4-C5, C5-C6 and prevertebral edema (arrow)

Blood work revealed no leukocytosis. Soft tissue neck X-ray showed reversal of the cervical lordosis, degenerative and erosive changes at C4-C5 and C5-C6, and thickening of the prevertebral soft tissues which are shown in Figure 1.

**Figure 2:**
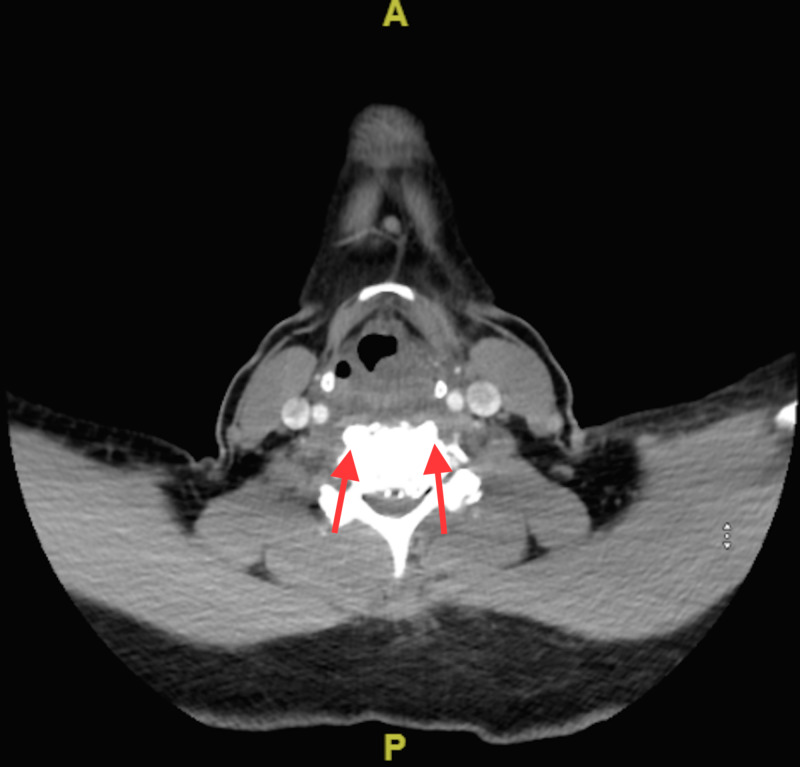
Axial view of CT cervical spine showing calcifications of the longus colli (arrows), appreciated on the anterior aspect of the vertebrae

**Figure 3:**
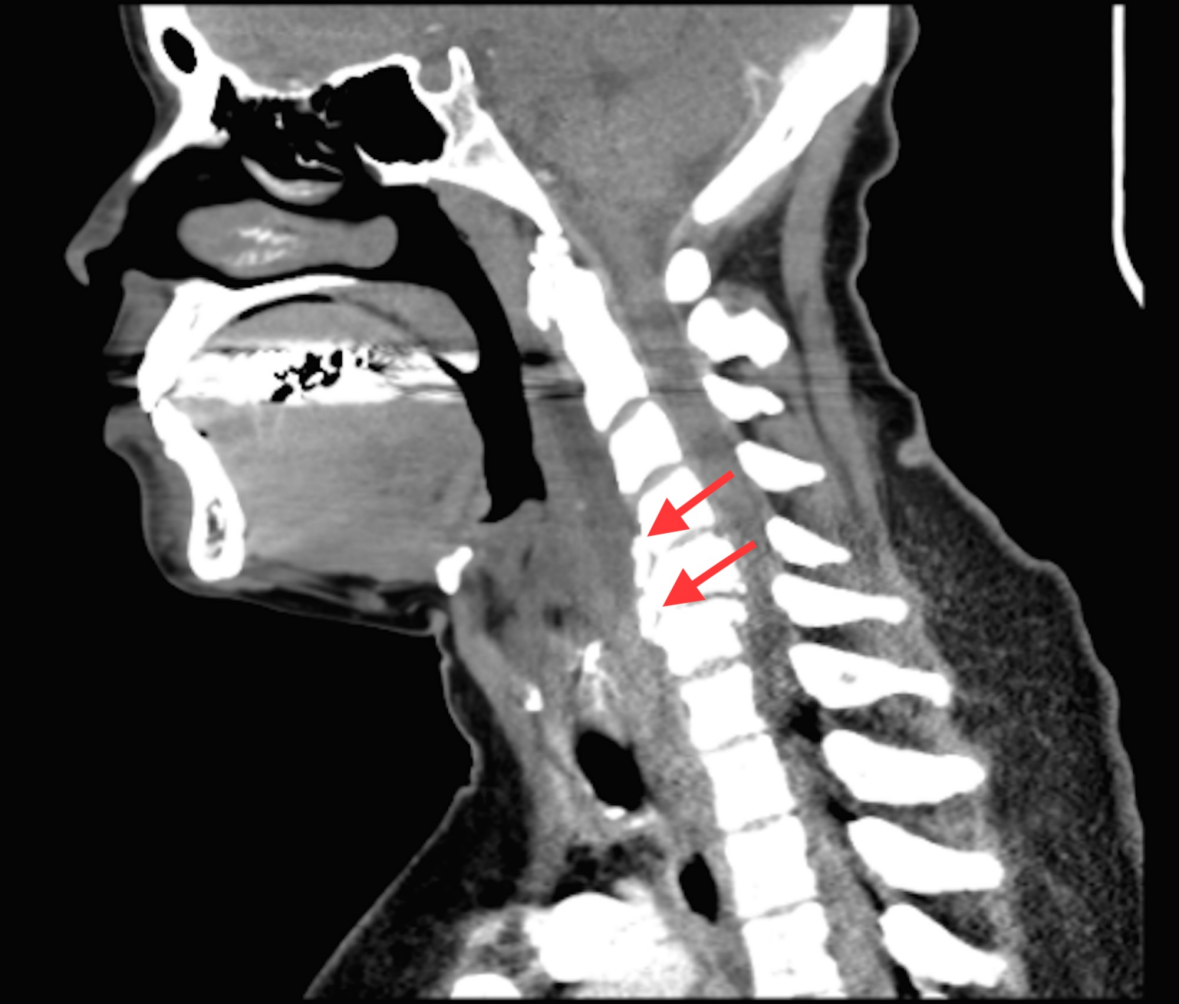
Sagittal view of CT cervical spine showing degenerative changes at C4-C5, C5-C6, calcific tendonitis of the longus colli (arrows)

She obtained a CT of the neck and cervical spine that showed osteophyte complexes at C4-C5 and C5-C6, as well as calcific tendonitis of the longus colli with a moderate amount of prevertebral free fluid (Figures 2, 3).

Neurosurgery was consulted from the ED who recommended getting an MRI of the neck while consulting ENT as well. ENT happened to be in the ED and performed a bedside laryngoscopy that showed edema of the left arytenoid with full functionality of the vocal cords and no signs of airway compromise. The patient was started on steroids, antibiotics, H2 blockers, and the ACE-inhibitor was discontinued due to suspected angioedema by the ENT. She was also admitted to the ICU for airway monitoring.

**Figure 4:**
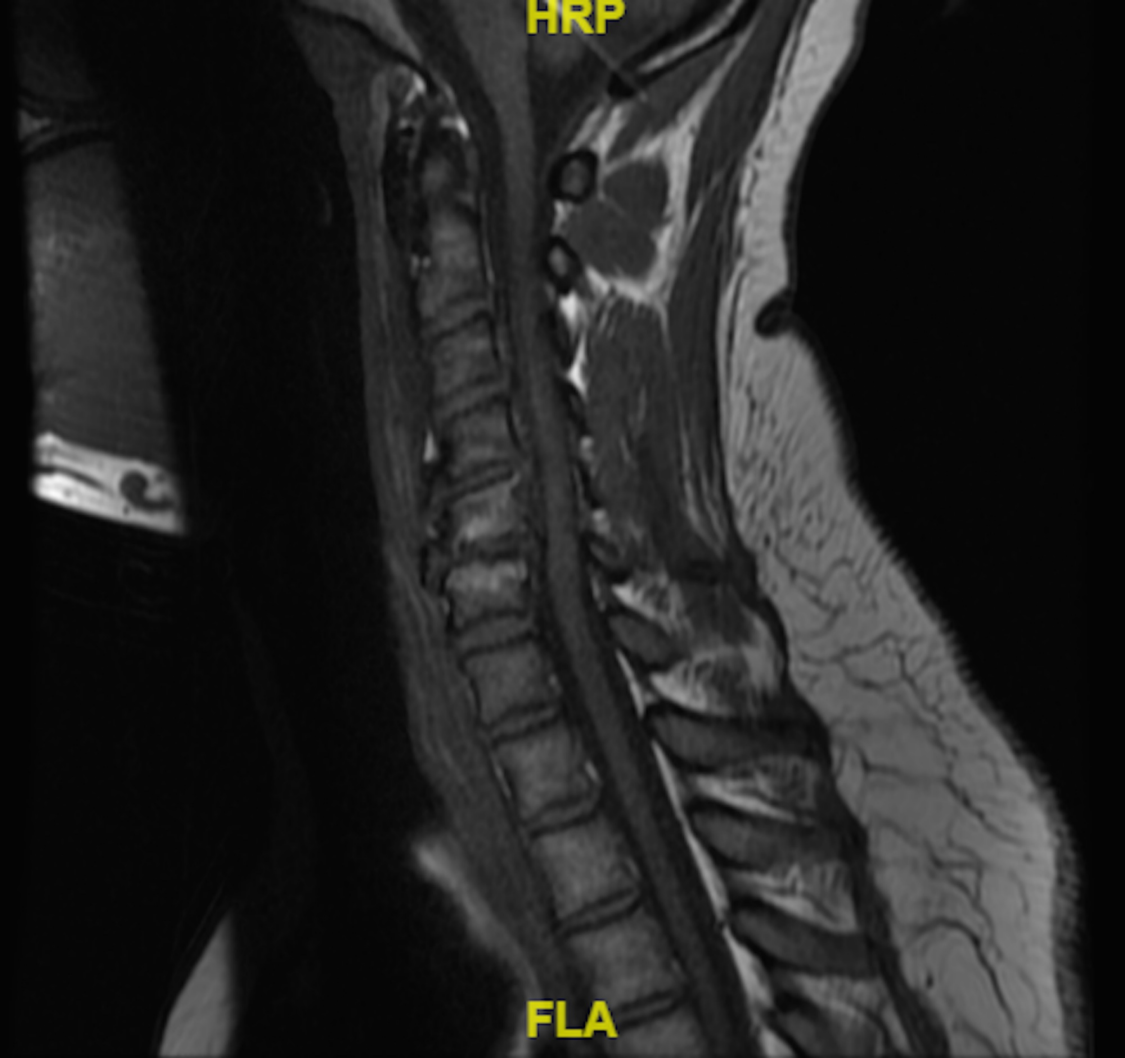
Sagittal view of MRI of the cervical spine. Note that the calcifications are not as well appreciated when compared to CT

The MRI of the neck again showed calcific tendonitis of the longus colli with moderate prevertebral fluid (Figure 4). Meanwhile, the patient had complete resolution of her symptoms in 24 hours and she was cleared from both neurosurgery and ENT to be discharged.

## Discussion

Acute calcific tendonitis is due to the deposition of calcium hydroxyapatite, which can cause significant pain and edema. Most commonly, this condition affects the shoulder, but can involve any part of the body. Demographically, acute calcific tendonitis can be seen in any age group, excluding the pediatric population. Age ranges have been documented to be as wide as 21-80 [5].

In terms of calcific tendonitis of the longus colli muscle, this condition is frequently misdiagnosed and continues to be a challenge, especially in the emergency department. These patients usually present with significant pain with other worrisome associated symptoms. The mechanism is poorly understood, but often includes previous history of trauma in association with pre-existing degenerative cervical spinal disease [6]. Due to the anatomy of the longus colli muscle, typical mechanisms of injury include hyperextension of the neck, such as whiplash from a motor vehicle accident.

Many diagnostic obstacles may arise when trying to diagnose acute calcific tendonitis of the longus colli muscle. Commonly, patients present with neck pain but can also complain of neck stiffness, occipital headache, decreased range of motion, sore throat, trismus, low-grade fever and odynophagia with associated leukocytosis and elevated inflammatory markers [7]. The differential diagnosis is diverse in etiology and severity, ranging from meningitis and retropharyngeal abscess to vertebral fracture and muscle strain.

The gold standard for diagnosis is computed tomography. CT of the neck can visualize the prevertebral edema in addition to the calcium deposits in the longus colli muscle. Visualization of the calcium deposits is the most radiologically sensitive technique to distinguish between a calcific tendonitis and retropharyngeal abscess [8]. CT has been shown to be superior to MRI, since MRI is unable to detect the calcium deposits, which is required to establish the diagnosis [8].

Prognosis is excellent for calcific tendonitis of the longus colli muscle. The condition is self-limiting and responds well to nonsteroidal anti-inflammatory drugs (NSAIDs) and supportive care. Patients have reported improvement of symptoms as early as 48 hours but can linger for up to two weeks [9]. In the ED, the key for providers is to reassure the patient. Patients may have significant symptomatology, but explaining the diagnosis, treatment plan and prognosis can allow the patient to feel secure about being discharged home.

In terms of our case, the patient had multiple reassuring qualities. Specifically, the patient was afebrile, non-toxic appearing, with no airway compromise, and without signs of angioedema. The labs were unremarkable for leukocytosis and CT imaging was able to confirm the diagnosis and exclude abscess formation. Despite this, the concerning aspect of the presentation was the acuity of onset and reported difficulty breathing. Due to this, multiple consultants were involved which led the patient through unnecessary fiberoptic scoping, IV antibiotics, MRI and ICU admission.

## Conclusions

Patients with acute calcific tendonitis of the longus colli muscle can be a challenge to diagnose. They often present with severe neck pain, with associated decreased range of motion, odynophagia, or even low-grade fever. White blood cell count and inflammatory markers can further muddy the diagnosis. For patients in which we are considering meningitis or retropharyngeal abscess, it is essential to obtain a thorough history and inquire about recent trauma, falls, or injuries, especially if the diagnosis is not clear. Soft tissue X-rays can demonstrate prevertebral edema and CT of the cervical spine can distinguish between abscess formation vs calcium deposits. Achieving the diagnosis can prevent the patient from being exposed to invasive procedures, IV antibiotics, and hospitalizations. It is essential for the provider to reassure the patient by explaining the diagnosis, treatment plan and prognosis. Involving the patient in a dialogue of how invasive procedures and admission are unwarranted can reassure the patient in the ED. The patient will improve with supportive care and NSAIDs.
